# Malignant Pleural Mesothelioma: Time Is Running Out

**DOI:** 10.3390/jcm10040648

**Published:** 2021-02-08

**Authors:** Filippo Lococo

**Affiliations:** 1 Università Cattolica del Sacro Cuore, 20123 Rome, Italy; filippo.lococo@policlinicogemelli.it; 2 Thoracic Surgery Unit, Fondazione Policlinico Universitario A. Gemelli IRCCS, 00168 Rome, Italy

Malignant pleural mesothelioma (MPM) is a rare but highly malignant disease of the pleura usually related to asbestos exposure. Although it is not officially accepted as an orphan disease, its low incidence imposes significant difficulties to physicians confronted with affected patients. Unlike other neoplasms which have benefited from recent advancements in technologies, new immune-oncological treatments and multiomic approaches (i.e., lung cancer), MPM seems not to have benefited from these new therapeutic frontiers and currently MPM-patients are doomed to a dismal prognosis (expected survival of 9–18 months after diagnosis, according to histological subtype) with substantially no real chance of care. Recently, an increase in interest emerged within the scientific community on this tumor because its incidence has dramatically increased in high income countries in the last years, following the prevision of various prediction models previously developed [1,2]. In 2010 the European Respiratory Society and the European Society of Thoracic Surgeons reported epidemiological projections that estimated an increasing incidence of MPM, with a peak around 2020 [3] and here we stand now with the same principal unsolved questions and substantially the same unsatisfactory therapeutic protocols. First, to early predict MPM in asbestos-exposed subjects is extremely hard. To date, according to the data available on MPM (prevalence, prognosis, and treatment) and the performance (sensitivity, specificity) of potential screening methods, the medical efficacy of large-scale screening is not recommended [3]. Several biological markers have been currently studied and further efforts are needed in this field of research [4].

Concerning the MPM management, the decision to select a treatment modality is currently determined by a few parameters (the stage of disease, histology, and the patient’s performance status) that are clearly inadequate to deal with the complexity of this tumor. Treatment for MPM remains a big challenge all over the world. Systemic chemotherapy has been shown to increase survival by a few months and the utility of immunotherapy shows promising preliminary results but its role in daily clinical practice is still far off being defined [5]. The role of surgery for MPM is very much debated, as a small, randomized study (MARS) has shown that the usefulness of radical surgery in the context of trimodality therapy is questionable, with no robust survival benefits and some concerns on the harm done [6].

By extremely simplifying the daily clinical practice, at this time MPM patients with clinical stage I–II epithelioid-MPM who are judged medically operable and surgically resectable may undergo multimodality therapy including surgery, while clinical stage-III/IV or non-epitheliod-MPMs (regardless of TNM-stage) are generally treated with systemic chemo(radio)therapy and/or best supportive care [7].

More complex prediction models using several clinical parameters have been evaluated in recent years [8] but these do not consider the biological features of MPM that probably have more influence than other parameters on the overall clinical course of the disease. We currently know that MPM stays as a heterogeneous entity in terms of morphology, transcription profile, and genetic landscape [9]. Asbestos which is the main risk factor for MPM significantly contributes to this heterogeneity, being the cause of a wide range of molecular aberrations [10] A significant number of studies investigated genetic and molecular profiles of MPM, and the expanding number of genomic data will probably help physicians in the therapeutic decision process in the near future [9,11]. However, the diagnostic and the therapeutic process have reached high levels of complexity, in an effort to take full advantage of different innovative variables, integrating a variety of different omics domains. The technologic improvements have brought out the biological and clinical complexity of this disease when facing the overwhelming number of variables to be considered in prevention, diagnosis, and treatment decisional processes. As in other neoplasms, such a multiomic approach will provide physicians with an enormous amount of data (“big data”) and the advent of artificial intelligence (AI) will play a decisive role in this.

All the above mentioned future perspectives are strongly encouraging but time is really running out. Indeed, as previously remarked, the peak of incidence is arriving (or is ongoing now) in high income countries. Second, but not yet relevant, we need to consider that in the not so far future MPM cases will dramatically increase in emerging middle-income countries (i.e., India). As reported in a recent study comparing the estimated global-level mesothelioma burden over the world [12], the disease burden estimated for lower-middle income countries, relative to those for high-income countries, has a larger effect on the global burden because of their population size. Since these very populous countries started their asbestos consumption in the late 90s with no significant restriction as today, it is reasonable to predict a global disaster in the coming years. In particular, three countries with large populations and known high asbestos use are likely to impact the global mesothelioma burden: China, Russia, and India [12]. Thus, research should be intensified both for an immediate effect on the high-income population and for future clinical practice applications in the low-income population.

In the present Special issue, we have aimed to offer to the readers an overview on the current evidence on MPM with particular emphasis on molecular epidemiology and new therapeutic frontiers.
